# Tolerability of facemask during physical exercises during COVID-19 pandemic: A systematic review

**DOI:** 10.4102/jphia.v16i2.610

**Published:** 2025-03-07

**Authors:** Akwagiobe F. Odey, Iwara I. Arikpo, Joshua Meremikwu, Mavis A. Otonkue, Nkwachukwu N. Chukwu, Freedman Ita-Lincoln

**Affiliations:** 1 Department of Paediatrics, Faculty of Clinical Sciences, University of Calabar Teaching Hospital, Calabar, Nigeria; 2 Department of Computer Sciences, Faculty of Science, University of Calabar, Calabar, Nigeria; 3 Cochrane Nigeria, Institute of Tropical Diseases Research and Prevention, University of Calabar Teaching Hospital, Calabar, Nigeria; 4 Department of Public Health and Community Medicine, University of Calabar Teaching Hospital, Calabar, Nigeria

**Keywords:** masks, during physical activity, prevention, SARS-CoV2, infection

## Abstract

**Background:**

Available evidence supports the use of facemasks by all groups to prevent respiratory infections, particularly severe acute respiratory syndrome coronavirus 2 (SARS-CoV-2). However, it is not clear whether these masks can be used safely during various intensities of physical exercise.

**Aim:**

To evaluate the impact of different types of facemasks on oxygen saturation, oxygen uptake, rate of perceived exertion and performance during different physical exercises.

**Setting:**

Healthcare databases.

**Method:**

We searched for articles published between 2020 to 05 September 2022. There was no restriction in age, language or setting. Electronic databases including The Cochrane Library - Central Register of Controlled Trials and Cochrane Database of systematic review and EPOC; MEDLINE; EMBASE were searched for articles for the period stated above. Risk of Bias in included studies was assessed using Cochrane risk of bias tool for RCTs.

**Results:**

Twenty-four randomised control trials with cross-over design were included. There was a total of 617 participants (373 males and 244 females). None of studies reported on prevention of SARS-CoV-2 infection by mask. The pooled effect estimate shows that wearing surgical mask did not affect oxygen saturation and oxygen uptake. Masks are tolerated during mild and moderate exercise, but reduce maximal exercise capacity. Children tolerate masks for submaximal exercise better than adults.

**Conclusion:**

During physical exercises, masks should be used with caution by healthy adults and children but should be avoided by elderly and ill patients.

**Contribution:**

This review offers current evidence on tolerability of facemask during physical exercises.

## Background

Severe acute respiratory syndrome coronavirus 2 (SARS-CoV-2) is a highly infectious disease that is estimated to have caused over 6.45 million deaths globally since the first report in late 2019.^1^ The virus is transmitted in humans from droplets expelled during coughing, sneezing and speaking as well as by contact with contaminated materials and surfaces.^2^ The incubation period for the virus ranges from 2 days to 5 days and can be spread from asymptomatic individuals, mildly symptomatic and individuals with full disease.^3,4^ Public health and social measures such as physical distancing and wearing of facemasks have been widely recommended to reduce the spread of the disease in the community.^2^

Facemasks act as a physical barrier preventing the spread of respiratory droplets. Available evidence suggests that mask-wearing reduces transmissibility per contact and the risk of acquiring SARS infection by up to 70%.^5^ A Cochrane systematic review on physical interventions to interrupt or reduce the spread of respiratory viruses reported that ‘overall, masks were the best-performing intervention across populations, settings and threats’.^6^

The level of protection provided by facemasks can vary across the different types available. Three types of facemasks meet essential parameters for protection and are recommended by the World Health Organization (WHO) for use in the community: non-medical masks, disposable medical masks and, where these are unavailable, well-fitting non-medical masks, including homemade multilayered masks. The WHO recommends disposable medical masks and non-medical masks for use by the public.^7^

The use of masks by the general public has potential advantages 0such as reducing the spread of potentially infectious aerosols or droplets from apparently healthy people as well as raising awareness of complementary transmission-preventing behaviours such as handwashing and not touching the eyes, nose and mouth. However, compliance with mask use is variable, and the improper use of masks reduces their protective effect and can result in headaches or breathing difficulties, facial irritation and difficulty with communication.

The WHO recommends wearing well-fitting masks that cover the nose and mouth in indoor settings where ventilation is poor, ventilation systems are inadequate or not properly maintained, and physical distancing of at least 1 m cannot be maintained to reduce the spread of SARS-CoV-2 infection in the community.^7^ This includes fitness centres and other places for physical activity or leisure.

The health and well-being of individuals has been the subject of concern and attention during the coronavirus disease 2019 (COVID-19) pandemic. Sedentary behaviour and low levels of physical activity are known to have negative effects on the health, well-being and quality of life of individuals. The WHO recommends 150 min of moderate-intensity or 75 min of vigorous-intensity physical activity per week or a combination of both. However, the pandemic has reduced opportunities to carry out physical exercises following the closure of fitness centres and self-isolation. In the wake of the pandemic, the rule of facemasks has been applied across the board, including when participating in physical activity indoors. Some studies have found that wearing the surgical mask reduced blood oxygen saturation significantly, leading to reduced performance and increased perceived exertion and stress.^8,9,10^ In contrast, other studies suggest that these effects may depend on the level of exertion, and facemasks have little effect in low- to moderate-intensity movement activities.^11,12,13^ Therefore, it is important to find a suitable balance between these important public health recommendations, such as improving and maintaining well-being with physical exercise while reducing the risk of transmission in the community.

### Objectives

To evaluate the effectiveness of wearing masks during physical activity for the prevention of SARS-CoV-2 infection.

To evaluate the tolerability of using a facemask during physical activity.

## Methods

### Characteristics of studies

The following characteristics were used to select studies for inclusion: randomised control trials (RCTs) on the use of facemasks or any type of covering during exercise on humans, on either male or female irrespective of the age (both children and adults) and setting.

### Eligibility criteria

#### Type of studies

We included only RCTs published between the years 2020 and 2022. There was no language restriction. Randomised control trials evaluating the effectiveness of any type of facemasks worn during physical activity that measured any of the outcomes of interest were included. All other study types including unpublished manuscripts and conference abstracts and proceedings were excluded.

#### Type of participants

We included all studies on adults and children in all settings that used facemasks during physical exercises.

#### Type of interventions

Interventions were the use of any type of mask (surgical or medical and respirators) or any other type of face covering during physical activity.

#### Type of comparisons

We compared the use of a facemask and/or protective face covering versus nonuse of facemask.

### Outcome measures

#### Primary outcomes

The primary outcome was SARS-CoV-2 infection.

#### Other outcomes

Measures of tolerability of mask during physical exercise, physiological measures (oxygen saturation, oxygen uptake, rate of perceived exertion) and exercise performance.

### Electronic searches

We searched the following databases: the Cochrane Library – Central Register of Controlled Trials (CENTRAL) July 2022 and Cochrane Database of Systematic Review 2005 to 31 August 2022; EMBASE Classic and EMBASE 1947 to 02 September 2022 and EPOC (Effective Practice and Organization of Care) for the period 2020 to 05 September 2022 of the definitive evidence search. We checked the reference lists of retrieved studies for additional reports of relevant studies although none was included. There were no language restrictions. We used the Preferred Reporting Items for Systematic Reviews and Meta-analysis (PRISMA) guideline and flow diagram to report the search and selection of studies.

### Data collection and analysis

*Selection of studies*: Five review authors (I.I.A., J.M., M.A.O., F.I.-L. and N.N.C.) independently screened the title and abstracts of identified studies. The identified studies were shared in five parts and double-screened by each of the five authors in rotation. A.F.O. screened approximately 20% of all the identified studies to ensure consistency in applying the eligibility criteria. The process was repeated during the review of the full text of the articles. Each of the five authors (I.I.A., J.M., M.A.O., F.I.-L. and N.N.C.) screened the same 5–10 articles to calibrate and test the developed review form. Once an agreement was reached, two authors (I.I.A. and N.N.C.) continued and screened the rest of the studies, while the other three authors (J.M., F.I.-L. and M.A.O.) screened the excluded full-text articles. Where there were multiple publications on the same study, we identified the studies using subscripts.

Any disagreements in this process were resolved in discussion with the lead author (A.F.O.) and the group editor (J.O.) or support author (O.O.). We presented a list of excluded studies and the reasons for their exclusion. Where applicable, we used Distiller^14^ software (https://www.distillersr.com/) to document this process.

### Data extraction and management

We extracted data onto a bespoke data extraction form developed for the review. The form was piloted independently by two review authors (A.F.O. and I.I.A.). Once an agreement was reached, the rest of the data extraction was conducted by six authors, with the lead author (A.F.O.) conducting quality assurance in a predefined number of included studies based on the overall number of included studies. Any disagreements in this process were resolved in discussion with the senior reviewer and editorial team (J.O., O.O. and M.M.).

For each study, we included background information on the location, the context of the study and any demographic information available. For each outcome, we aimed to extract the number of participants randomised or included in the study as well as the number analysed in each arm or group. For dichotomous outcomes, we recorded the number of participants experiencing the event and the number assessed in each arm or group. For continuous outcomes, we extracted arithmetic means and standard deviations for each intervention group, together with the numbers assessed in each group.

### Extracted data items

Data were extracted on the following outcomes: prevention of transmission of SARS-CoV-2 during physical exercises, oxygen saturation, oxygen uptake in millilitres/minute, exercise performance and perceived exertion. The aim was to extract all listed outcomes from each included study. However, not all studies reported on all the outcomes, but all outcomes reported were extracted from the included studies.

### Assessment of risk of bias in included studies

We used the Cochrane Risk of Bias tool for RCTs. We assessed seven components: random sequence generation; allocation concealment; blinding of participants and personnel; blinding of outcome assessment; incomplete outcome data; selective outcome reporting and other biases. Judgement of ‘yes’, ‘no’ and ‘unclear’ was made to indicate a low, high or unclear risk of bias. We presented the results of the assessment in a ‘Risk of Bias’ summary form. Three review authors independently assessed the risk of bias in each included study using a ‘Risk of Bias’ form. We attempted to contact the study authors if the necessary information was not specified or was unclear. We resolved any disagreements by discussion between review authors.

### Measures of treatment effect

The outcomes of interest were infection with SARS-CoV-2, oxygen saturation, oxygen uptake in millilitres/minute, exercise performance and perceived exertion. We planned to use adjusted measures as the primary effect measures when available and indicated what factors were adjusted for. We extracted the effects estimates of mean differences (MD) and standard deviation (s.d.) for each outcome and calculated the 95% confidence intervals (CIs) of the MDs using a generic inverse variance. All included studies were of a cross-over design; we assessed whether estimates were properly adjusted to account for clustering effects.

### Assessment of heterogeneity

We assessed statistical heterogeneity using the *I*^2^ statistic. We considered heterogeneity statistically significant when the *I*^2^ statistic was ≥ 50%. We used the random-effects model for meta-analysis when the *I*^2^ statistic was > 50%.

### Data synthesis

#### Subgroup analysis and investigation of heterogeneity

In the absence of substantial clinical or methodological heterogeneity (Chi-squared test with a *p*-value of 0.10 or *I*^2^ statistic ≥ 50%), we pooled the data for a meta-analysis, using risk ratios (RR) with 95% CIs and a random-effects model. We presented the results for continuous outcomes as MD and time-to-event outcomes as hazard ratios, all with 95% CIs. Forest plots were used to summarise the findings from the included studies. If the included studies were not sufficiently homogeneous to combine in a meta-analysis, we displayed the results of included studies in a forest plot but suppressed the pooled estimate.

We combined data from studies that have similar designs, interventions and outcome measures. We decided whether to use a fixed- or random-effects model based on the consideration of clinical and methodological heterogeneity between studies. We stratified analyses by study design (i.e. cross-over RCTs) and participants (athletes or non-athletes). We only conducted a meta-analysis if we identified enough studies with both outcome indicator estimates and a measure of precision.

The offline version of Cochrane Review Manager 5 (REVMAN 5) was used to set up and manage these comparative effectiveness reviews, perform a meta-analysis and generate forest plots. Where a meta-analysis was not possible for comparative effective studies, we presented a narrative summary of the data based on the Synthesis without meta-analysis in systematic reviews (SWiM) guidelines.^15^ Efforts were made to reach authors of articles with missing data by email. Where it was not possible, only available data were analysed.

### Assessment of quality of evidence

We assessed the quality of the evidence using the Grading of Recommendations Assessment, Development and Evaluation tool (GRADE) approach. We appraised the quality of evidence for each outcome against five criteria: risk of bias (an appraisal of the overall risk of bias for trials contributing to the outcome); consistency (an evaluation of explained and unexplained heterogeneity); directness (an appraisal of how directly the included trials address the review question); precision (an assessment of the statistical precision of the result) and publication bias (an assessment of the risk of publication bias). Certainty of evidence started at high quality, but we downgraded it to moderate, low or very low for the following reasons: limitations in study design or execution (risk of bias), inconsistency of results, indirectness of evidence, imprecision or publication bias. The summary of evidence for surgical mask compared with no mask is presented in Table 2. The GRADE evidence for respirators versus no masks is presented in Table 3.

**TABLE 1:** Study characteristics of included studies.

Study	Participant number (*n*)	Age (mean ± s.d.; in years)	BMI (mean ± s.d.)	Gender		Protocol (intensity categorisation)	Type of mask (surgical/medical, N95/FFP2, cloth)	Mean ± s.d. of mask			
				Male	Female			Oxygen saturation %	Oxygen uptake in mL/min	Exercise performance	Perceived exertion/RPE
Alkan et al. (2022)^16^	26	37.35 ± 15.99	26.10 ± 4.96	11	-	Maximal exercise test on a treadmill to exhaustion. Bruce protocol was used to determine maximal exercise capacity	No mask Surgical No mask Surgical	96.18 ± 1.54† 95.82 ± 2.36† 97.73 ± 0.80‡ 97.40 ± 1.76‡	- - - -	- - - -	5.00 ± 1.84† 5.27 ± 2.05† 5.53 ± 2.13‡ 6.13 ± 2.03‡
Amput et al. (2022)^17^	-	69.60 ± 3.46	21.14 ± 1.26	25	15	6 min walking test (6-MWT)	No mask Surgical Cloth	97.50 ± 1.09 97.68 ± 1.16 97.83 ± 1.15	- - -	- - -	7.03 ± 1.21 8.25 ± 1.66 7.35 ± 1.23
Cabanillas et al. (2021)^18^	50	20.96 ± 5.36	-	26	-	6-MWT based on the recommendations of the American Thoracic Society	No mask Surgical mask FFP2	95.88 ± 4.74 94.88 ± 8.22 96.94 ± 2.60	- - -	- - -	1.54 ± 1.69 2.48 ± 1.71 3.52 ± 1.97
Dalakoti et al. (2022)^19^	50	31.7 ± 6.5	23.0 ± 3.1	27	23	Bruce protocol on an exercise treadmill	No Mask Surgical	93.1± 5.8 92.1 ± 6.7	- -	- -	7.1 ± 2.2 7.5 ± 1.8
Darnell et al. (2022)^20^	12	20.1 ± 1.2	-	-	6	Modified Astrand Running Protocol for maximal treadmill exercise	No mask Cloth N95	(-0.47, SE 0.09) -0.58, SE 0.09, -0.64, SE 0.08,	- - -	- - -	1.59, SE 0.08) 1.91, SE 0.05; 1.79, SE 0.06,
Driver et al. (2022)^8^	31	-	-	-	14	CPET following Bruce protocol: 21 min (3 min walking warm-up and 6 CPET stages of 3 min each)	No mask Cloth	95.1 ± 2.4† 93.4 ± 3.1§	43.9 ± 8.1 32.2 ± 9.0	- -	5.5 ± 2.3 (7.2 ± 2.9)
Egger et al. (2021)^12^	-	27 ± 7	22.5 ± 1.8	16	-	Incremental exercise tests on bicycle ergometer	No mask Surgical FPP2	- - -	58.8 ± 5.7 45.0 ± 10.2 47.6 ± 8.5	- - -	5.1 ± 0.5 4.8 ± 0.4 4.9 ± 0.5
Fikenzer et al. (2020)^9^	-	38.1 ± 6.2	24.5 ± 2.0	12	-	Cycle ergometer semi-recumbent graded until volitional exhaustion; vigorous intensity	No mask Surgical FFP2/N95	- - -	39.7 ± 5.8 37.9 ± 6.0 34.5 ± 5.3	277 ± 45.9 269 ± 45.1 263 ± 41.7	2.8 ± 2.2 5.2 ± 2.1 7.0 ± 1.7
Fukushi et al. (2021)^21^	24	-	-	-	9	Bruce treadmill protocol: 15 min (3 min walking warm-up and 4 walking stages of 3 min each)	No mask Cloth Surgical	96.5 (93.75–97) 96 (93.75–97) 96 (94.75–96)	- - -	- - -	5 (3.75–6) 6 (5–7.25) 6.5 (6–9)
Kogel et al. (2021)^36^	12	63.8 ± 12	-	12	-	Three incremental cardiopulmonary exertion tests were performed on a semi-recumbent ergometer	No mask Surgical FFP2	- -	- -	- -	- -
Lin et al. (2022)^22^	-	23.4 ± 3.6	21.7 ± 3.7	18	16	CPET incremental exercise stage until voluntary exhaustion	No mask Surgical	- -	24.2 ± 5.9 22.3 ± 6.1	- -	- -
Marticorena et al. (2023)^23^	9	13 ± 1.0	18.4 ± 2.1	-	6	Progressive square-wave test at four intensities	No mask Cloth	- -	- -	- -	- -
NG et al. (2022)^24^	8	24.5 ± 3.3	23.6 ± 2.4	-	-	Graded cycling test on a Lode Excalibur bicycle until exhaustion	No mask Surgical Taped filter mask	92 ± 3 90 ± 6 89 ± 4	- - -	278 ± 56 269 ± 56 247 ± 56	9.8 ± 0.5 9.7 ± 0.7 9.8 ± 0.5
Pimenta et al. (2021)^33^	12	29.8 ± 5.3	22.1 ± 2.4	8	-	Bruce treadmill protocol	No mask Surgical Respirator	94.5 ± 2.7 92.5 ± 3.9 91.3 ± 4.0	- - -	15.3 ± 1.7 16.4 ± 2.4 17.1 ± 2.4	- - -
Poncin et al. (2022)^34^	28	-	-	-	9	1 min sit-to-stand test (STST)	No mask Surgical	93.7 ± 2.5 93.9 ± 2.2	- -	- -	3.6 ± 2.9 (5.1 ± 2.9)
Poon et al. (2021)^25^	-	21.9 ± 1.4	21.6 ± 2.1	7	6	Continuous, incremental, graded uphill treadmill running test to volitional exhaustion	No mask Surgical	97.5 ± 0.8 97.0 ± 1.4	- -	- -	14.2 ± 2.1 15.5 ± 1.5
Reychler et al. (2021)^26^	-	22 ± 1.6	22.1 ± 2.8	9	11	Borg Cr10 scale (1 min sit-to-stand test)	No mask Surgical Cloth	98 (97–98) 98 (97–98) 97 (97–98)	- - -	- - -	3 [1.25; 4] 3 [2; 4] 3 [2.5; 4]
Reychler et al. (2022)^27^	37	8.5 (8–9); 10 (10–11)	-	16	-	1 min sit-to-stand tests using modified Borg and Dalhousie scales	No mask Surgical No mask Surgical	98 (96–100)§ 98 (97–100)§ 98 (94–100)¶ 98 (97–99)¶	- - - -	40.5 (22–63)§ 42.5 (30–63)§ 38 (31–54)¶ 39 (22–46)¶	0.5 (0–3)§ 2 (0–4) § 0.5 (0–4)¶ 2 (0.5–6)¶
Rosa et al. (2022)^28^	-	27.5 ± 4.4	-	12	-	Protocol: maximum strength test, using one maximum repetition protocol	No mask FFP2/N95	97 (95.25–98) 97 (96–98.75)	- -	- -	8 (7.0–9.0) 7.5 (6.2–8.0)
Salles-Rojas et al. (2021)^35^	77	44 ± 12	-	49	-	6-MWT	No mask Surgical (*n* = 36) N95 (*n* = 41)	- 90.3 ± 3.5 90.5 ± 3.7 89.4 ± 4.3 89.4 ± 4.3	- - -	- - -	- 1.8 ± 2.2 1.8 ± 1.2 2.4 ± 2.0 2.3 ± 2.0
Shaw et al. (2020)^29^	14	28.2 ± 8.7	-	-	7	Cycle ergometer 10.3 (2.35) min; until volitional exhaustion, vigorous intensity	No mask Surgical Cloth	96 ± 4 96 ± 3 95 ± 3	- - -	- - -	9.9 ± 0.5 9.9 ± 0.4 9.7 ± 0.6
Shaw et al. (2021)^30^	26	11.7 ± 1.6	-	21	5	Cycle Ergometer using Wingate test	No (Sham) mask Surgical	- -	- -	- -	- -
Shui et al. (2022)^31^	12	34 ± 4	21 ± 3	6	-	Three incremental exercise tests in participants with no mask, surgical and N95 masks using computer-controlled bicycle ergometer	No mask Surgical N95	- - -	1653 ± 401 1345 ± 325 1417 ± 363	- - -	- - -
Steinhilber et al. (2022)^32^	39	38.2 ± 14.2	23.7 ± 2.4	20	-	Physical working capacity (PWCmax and PWCsubmax) tests	No mask Surgical FFP2 Community mask	97.30 ± 3.74 97.42 ± 3.38 98.29 ± 2.94 97.90 ± 4.03	- - - -	1.91 ± 0.70 1.84 ± 0.72 1.74 ± 0.65 1.83 ± 0.70	3.51 ± 1.25 3.80 ± 1.67 3.59 ± 1.83 3.42 ± 1.39

Note: Please see the full reference list of the article Odey AF, Arikpo II, Meremikwu J, Otonkue MA, Chukwu NN, Ita-Lincoln F. Tolerability of facemask during physical exercises during COVID-19 pandemic: A systematic review. J Public Health Africa. 2025;16(2), a610. https://doi.org/10.4102/jphia.v16i2.610, for more information.

M, male; F, female; BMI, body mass index; s.d., standard deviation; RPE, rate of perceived exertion; CPET, Cariopulmonary exercise test; MWT, 6-minute walk test; CPET, cardiopulmonary exercise test.

† , male;

‡ , female;

§ , 8–9 years;

¶ , 10–11 years.

**TABLE 2:** GRADE evidence profile: Surgical masks compared to no masks for health problems or populations.

Number of studies	Certainty assessment						Number of patients		Effect		Certainty
	Study design	Risk of bias	Inconsistency	Indirectness	Imprecision	Other considerations	Surgical masks	No mask	Relative (95% CI)	Absolute (95% CI)	
**Oxygen saturation**											
6	Randomised trials	Serious†	Not serious	Not serious	Serious‡	None	149	149	-	MD **0.61** (1.32 to 0.11)	⨁⨁◯◯ Low
**Oxygen uptake**											
3	Randomised trials	Serious†	Serious§	Not serious	Serious¶	None	62	62	-	MD **5.49** (12.16 to 1.17)	⨁◯◯◯ Very low
**Exercise performance**											
4	Randomised trials	Serious†	Not serious	Not serious	Serious††	None	57	57	-	MD **0.22** (0.47 to 0.03)	⨁⨁◯◯ Low
**Rate of perceived exertion**											
7	Randomised trials	Serious†	Serious‡‡	Not serious	Very serious§§	None	168	168	-	MD **0.48** (0.01 to 0.94)	⨁◯◯◯ Very low

CI, confidence interval; MD, mean difference; GRADE, Grading of Recommendations Assessment, Development and Evaluation tool.

† , Downgraded by one for risk of bias because of serious concerns about the variability in the washout periods in the included studies;

‡ , Downgraded by one for imprecision: Confidence interval includes potential benefit and harm and optimal information size not attained;

§ , Downgraded by one for inconsistency: The *I*^2^ test for statistical heterogeneity is equal to 86%;

¶ , Downgraded by one for imprecision: Confidence interval includes potential benefit and harm and optimal information size not attained;

†† , Downgraded by one for imprecision: Optimal information size not met as only 57 participants contributed to the meta-analysis;

‡‡ , Downgraded by one for inconsistency: The *I*^2^ test for statistical heterogeneity is equal to 62%;

§§ , Downgraded by one for imprecision: Optimal information size not met as only 168 participants contributed to the meta-analysis.

**TABLE 3:** GRADE evidence profile: Respirator compared to none for health problem or population.

Number of studies	Certainty assessment						Number of patients		Effect		Certainty
	Study design	Risk of bias	Inconsistency	Indirectness	Imprecision	Other considerations	Respirator	none	Relative (95% CI)	Absolute (95% CI)	
**Oxygen saturation**											
2	Randomised trials	Serious†	Serious‡	Not serious	Serious§	None	62	62	-	MD **0.91** (5.07 to 3.25)	⨁◯◯◯ Very low
**Oxygen uptake**											
2	Randomised trials	Serious†	Serious¶	Not serious	Serious††	None	28	28	-	MD **8.08** (13.96 to 2.21)	⨁◯◯◯ Very low
**Exercise performance**											
1	Randomised trials	Serious†	Not serious	Not serious	Serious‡‡	None	12	12	-	MD **14** (49.09 to 21.09)	⨁⨁◯◯ Low
**Perceived exertion/RPE**											
3	Randomised trials	Serious†	Serious§§	Not serious	Serious¶¶	None	78	78	-	MD **1.88** (0.3 to 4.06)	⨁◯◯◯ Very low

CI, confidence interval; MD, mean difference; RPE, rate of perceived exertion; GRADE, Grading of Recommendations Assessment, Development and Evaluation tool.

† , Downgraded by one for risk of bias: Serious concerns about the variability in the washout periods in the included studies;

‡ , Downgraded by one for inconsistency: The *I*^2^ test for statistical heterogeneity is equal to 86%;

§ , Downgraded by one for imprecision: Optimal information size not met as only 62 participants contributed to the meta-analysis. Confidence interval includes appreciable benefits and harm;

¶ , Downgraded by one for inconsistency: The *I*^2^ test for statistical heterogeneity is equal to 68%;

†† , Downgraded by one for imprecision: Optimal information size not met as only 28 participants contributed to the meta-analysis;

‡‡ , Downgraded by one for imprecision: Optimal information size not met as only 12 participants contributed to the meta-analysis;

§§ , Downgraded by one for inconsistency: The *I*^2^ test for statistical heterogeneity is equal to 96%;

¶¶ , Downgraded by one for imprecision: Optimal information size not met as only 78 participants contributed to the meta-analysis.

### Ethical considerations

All procedures performed in this secondary research study followed all international, national, and/or institutional guidelines. As a secondary research study, ethical clearance was not required.

## Results

**FIGURE 1:**
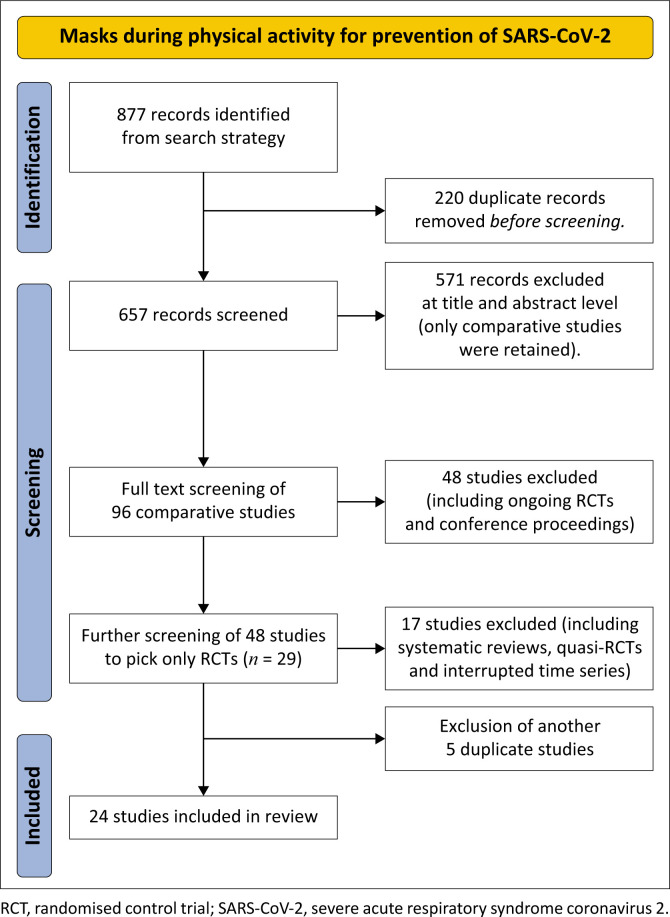
Profile of included studies.

Our search strategy yielded 877 studies. After identifying and removing duplicates, 657 remained. We screened the titles and abstracts of the studies, removed all noncomparative studies and were left with 92 comparative studies. We carried out full-text screening of all comparative studies, removed ongoing RCTs and conference proceedings, and were left with 48 studies. After further full-text screening, we included 24 RCTs (Figure 1).

## Participants

The 24 included studies had a total of 617 participants, with 373 males and 244 females. The mean age for the study participants was 30.68 years (the mean age of males was 30.01 years while that of females was 30.16 years). The participants in the included studies included children, adolescents, adults and the elderly. All 24 included studies were RCTs with a cross-over design and compared one or more types of masks (surgical/medical masks, N95/FFP2, cloth masks or other filtering devices) against control (no masks).

Twenty-one of the included studies^8,9,12,16,17,18,19,20,21,22,23,24,25,26,27,28,29,30,31,32^ were on healthy participants, 17 of which were on adults 18 years and above, three were on children/teenagers between 8 years and 16 years^23,27,30^ while one study was on elderly participants ≥ 65 years.^17^ Six of the studies on healthy volunteers were on health/medical workers.^9,19,22,26,31,33^ Three of the studies were on hospitalised patients, two of the participants were recovering from COVID-19,^34,35^ while one was on patients with chronic heart failure that was under spiroergometry with or without masks.^36^

### Characteristics of included studies

The characteristics of the included studies (participant characteristics and protocol used), with a focus on the descriptive summary statistics for each group of the included studies, are displayed in Table 1. The table shows the surname of the first author, year of publication, participant characteristics (number, gender and age), protocol followed for exercise, type of mask used and physiologic variables.

### Risk of bias and criteria of methodological quality

The risk of bias and methodological quality of the included studies were calculated following the Cochrane criteria using Review Manager 5.4.1. A.F.O. entered the data, and this was reviewed by J.O. and O.O.; any differences were resolved through discussion. Figure 4 shows assessment of risk of bias for included studies.

### Type of mask used

Different types of masks were used in the studies. Seven studies used surgical masks alone,^16,19,22,25,27,30,34^ another seven used surgical masks in combination with N95/FFP2^9,12,18,31,33,35,36^ and one study used surgical and tapered filter masks.^24^ Cloth masks were used alone in two studies,^8,23^ in combination with surgical masks in four studies^17,21,26,29^ or in combination with N95 in one study.^20^ One study used only N95/FFP2,^28^ while another study used surgical, FFP2 and community masks.^32^

### Use of surgical masks in normal healthy adults

A meta-analysis of studies that compared physiological properties between surgical masks and no masks was carried out among healthy participants during different exercises. Analysis was done on four parameters, namely oxygen saturation, oxygen uptake, exercise performance and rate of perceived exertion.

### Surgical masks: Oxygen saturation among healthy participants

**FIGURE 2:**
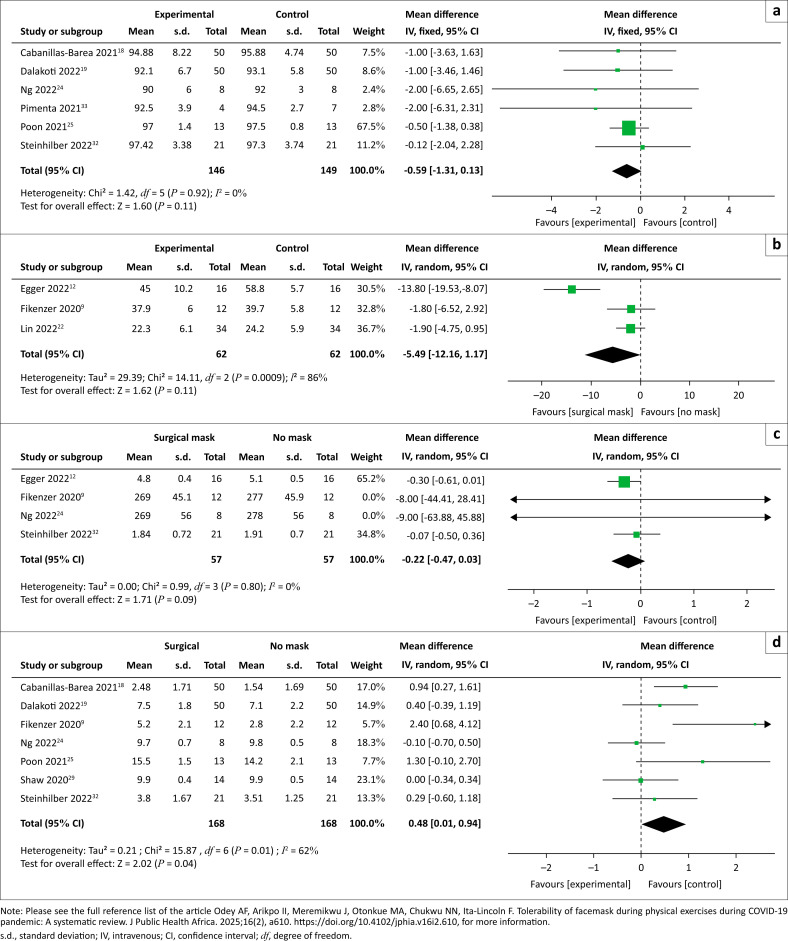
(a) Oxygen saturation in participants in surgical masks versus no mask, (b) oxygen uptake in included studies, (c) exercise performance in surgical masks versus no mask and (d) rate of perceived exertion in included studies.

Ten studies reported outcomes on oxygen saturation, but data could only be combined in six studies.^18,19,24,25,32,33^ Figure 2(a) shows the pooled effect estimate (six effect sizes), with MD = –0.59 (95% CI: 1.31, 0.13); *I*^2^ = 0% showing that there is no difference between wearing a surgical mask and no mask with regards to oxygen saturation.

### Surgical masks: Oxygen uptake among healthy participants

Five studies reported on oxygen uptake^8,9,12^ in a healthy population who performed various types of exercises, but data for meta-analysis were extracted from three studies.^9,12,22^ Figure 2(b) shows a meta-analysis of the data extracted from the studies and shows that there was no difference between participants who wore surgical masks and those who did not; MD was −5.49 (95% CI: –12.6, 1.17); *I*^2^ = 86%. The other study that reported on oxygen uptake was by Shui et al. (2022),^31^ but data could not be pooled into the meta-analysis. The study showed that wearing a surgical mask leads to a negative impact on cardiopulmonary function, and the effect is more pronounced with an N95 mask and suggested that attention should be paid to exercise while wearing surgical or N95 masks.

### Surgical masks: Exercise performance in included studies

To assess exercise performance, data were extracted from four studies for inclusion in the meta-analysis of participants who wore surgical masks versus those who wore no masks.^9,12,24,32^ Figure 2(c) shows that there was no difference between participants who wore surgical masks and those who did not wear masks (MD = –0.22 [95% CI: –0.47, 0.03]; *I*^2^ = 0%). The study by Egger et al. (2022)^12^ further reported that taped filter masks are tolerated well during mild and moderate exercise but reduce maximal exercise capacity when compared to no mask.

### Surgical masks: Rate of perceived exertion in included studies

A total of seven studies reported the effect of surgical masks on the rate of perceived exertion among the healthy adult population.^9,18,19,24,25,27,32^ The result of the meta-analysis revealed a significantly lower rate of perceived exertion among participants who did not wear surgical masks (MD = 0.48 [95% CI: 0.01, 0.94]; *I*^2^ = 62%). Three studies^9,18,33^ further reported that participants who wore respirator (FFP2 or N95) masks had a higher rate of self-perceived exertion with FFP2 or N95 than those who used surgical masks.

### Study outcomes in participants who used respirators

**FIGURE 3:**
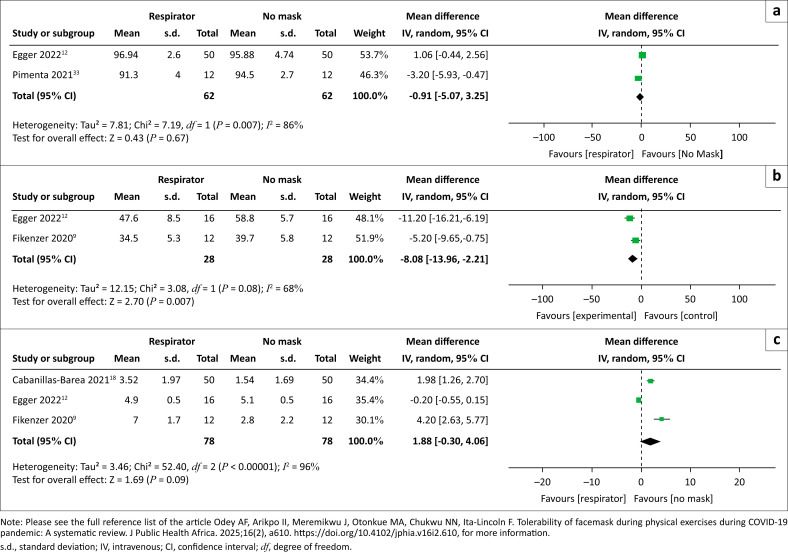
(a) Oxygen saturation in respirator masks, (b) oxygen uptake among respirator users and (c) rate of perceived exertion.

Three types of respirator masks (N95, FFP2, and taped filter mask) were used in the included studies. Two studies^12,33^ contributed data to oxygen saturation among healthy adults engaged in various levels of exercise. Figure 3(a) shows that there was no difference between those who wore respirator masks and those who did not wear them (MD = –0.91 [95% CI: –5.07, 3.25]; *I*^2^ = 86%).

**FIGURE 4:**
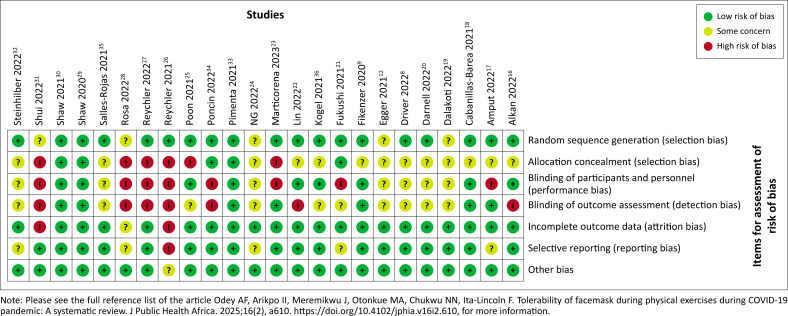
Risk of bias assessment in included studies.

### Use of respirators (N95 and FFP2): Oxygen uptake in participants

Oxygen uptake among participants who used respirators was assessed in two studies.^9,12^ Meta-analysis of the data from the two studies showed that oxygen uptake was significantly reduced among those who wore respirator masks; MD = –8.08 (95% CI: –13.96, –2.21); *I*^2^ = 68%.

### Use of respirators (N95 and FFP2): Exercise performance

Only one study reported on the performance of exercise using a respirator mask.^9^ The study concluded that exercise performance was highly impaired by FFP2 and N95 facemasks in healthy individuals.

### Use of respirators (N95 and FFP2): Rate of perceived exertion

A total of three studies^9,12,18^ contributed data to the analysis of the rate of perceived exertion among participants who used respirators during exercises. Figure 3(c) shows that there was no difference between those who wore respirators and those who wore no masks (MD = 1.88 [95% CI:–0.30, 4.06]; *I*^2^ = 96%). Two of the studies included in the meta-analysis^8,18^ reported that participants who wore respirator (FFP2 or N95) masks had a higher rate of self-perceived exertion with FFP2 or N95 than those who used surgical masks. On the other hand, participants in the study by Egger et al. (2022)^12^ reported less exertion with FFP2 than with surgical masks. However, when the data were merged, no effect was noticed.

### Effect of facemasks on children

Three studies examined the effect of facemasks on a total of 72 children between the ages of 8 and 16 years.^23,27,30^ Two of the studies used surgical masks, while the third used triple-layered cloth masks. The first study by Reychler et al. (2022)^27^ evaluated the effect of surgical masks on 37 healthy children 8–12 years of age using the 1 min sit-to-stand submaximal exercise test. The results showed that surgical facemasks had no impact on exercise performance in healthy children.^29^ The second study by Shaw et al. (2021)^30^ evaluated 26 youth hockey players, in the age group 10-13 years wearing surgical masks on cycle ergometers. Results showed that wearing a facemask had no effect on moderate exercise performance in young hockey players.^30^ The third study on children by Marticorena et al. (2022)^23^ enrolled nine healthy, active children between 11 years and 16 years of age. The authors analysed the use of triple-layered cloth masks in a progressive square-wave test at four intensities of moderate, heavy, very heavy and severe domains. The results showed that even with heavy- and severe-intensity exercise, perceived exertion and respiratory functions were within tolerable levels with triple-layered facemasks.

### Studies on ill patients

Two studies evaluated the effect of the use of masks during exercise on survivors of COVID-19,^34,35^ and one study was on elderly patients with chronic heart failure.^36^ The first study on COVID-19 patients by Salles-Rojas et al.^35^ studied the effects of either surgical masks or N95 masks on 77 patients 30 days after being discharged from the hospital for COVID-19. The participants performed a 6 min walking test twice, either wearing surgical or N95 masks. The results showed that there was no difference between those who wore masks (surgical or N95) and those who did not wear any mask. The second study was conducted on 28 adult patients who survived COVID-19 close to discharge from the hospital.^34^ The study evaluated the impact of surgical masks on submaximal exercise performance and reported that although there was increased dyspnoea while wearing a mask at rest, there was no impact on submaximal exercise performance. Both studies suggest that survivors of COVID-19 can safely use facemasks for submaximal exercise performance.

The third study was on the use of facemasks (surgical and FFP2) using spiroergometry to test exercise performance on 12 chronic heart disease elderly patients.^36^ The study showed that both surgical and FFP2 masks reduced exercise capacity in heart failure patients, use of FFP2 reduced oxygen uptake as well. Thus, care should be taken in using any type of mask on elderly chronic heart failure patients.

### Mask use in elderly participants

One study evaluated the use of facemasks (surgical and cloth) during exercise on 40 elderly patients above 68 years of age.^17^ Participants were assessed at rest and after performing a 6 min walk test wearing masks, and the results were compared with controls. Results showed that cloth masks and surgical masks did not impact oxygen saturation, but there was a significant increase in perceived exertion in those who wore surgical masks and cloth masks after performing a 6 min walk test.

## Discussion

This systematic review and meta-analysis have shown that among healthy adult populations, physiological measures (oxygen saturation and oxygen uptake) are not significantly affected during the conduct of mild to moderate exercises while wearing either a surgical mask or respirator mask (N95 or FFP2). However, the use of facemasks impacted the performance of high-intensity exercises among athletes or healthy young adults.^12,20,21,24^ Hence surgical masks can be used safely by healthy adults for mild to moderate physical activity. For psychological measurements in healthy adult participants, meta-analysis suggests that the rate of perceived exertion is significantly higher among participants who wore surgical masks compared to those who did not wear surgical masks. One of the studies not included in the meta-analysis reported that exercise performance was impacted by the use of either N95 or cloth masks.^20^ Meta-analysis of the perceived exertion in included studies shows that most participants perceived more exertion while wearing any type of facemask compared to the controls. This effect was less common among wearers of surgical masks compared to respirator masks (N95 or FFP2). Based on the rate of perceived exertion, it would be reasonable to suggest that masks should not be worn during rigorous exercises. This will be in keeping with the WHO recommendation that respirator masks such as N95, NK95, FFP2 or filtered masks should be used with caution during physical exercises.

A review of the use of masks during physical exercises in children suggests that surgical masks had no impact on dyspnoea, cardiorespiratory performance and exercise during submaximal exercise in healthy children.^23,27^ Specifically, facemasks could be worn by youth hockey players without negative consequences on performance, especially where vaccination protocols are not available to children.^30^ In developing countries like Nigeria, COVID-19 vaccination is concentrated in the more vulnerable groups, such as the elderly and others with comorbidities. Vaccination of children is not a priority in most developing countries.

For the use of facemasks during exercises among patients after treatment for COVID-19, both physiological parameters and perception of exhaustion were suboptimal shortly after discharge from the hospital but improved 1 month later.^35^ It would appear that allowing sufficient time for the body to recover could ensure the ability of patients to tolerate masks for mild to moderate exercises. For elderly patients with chronic heart failure, all parameters were adversely affected by the use of any type of facemask.^36^ Thus, facemasks should be avoided in elderly patients with chronic heart failure for any form of exercise.

The certainty of evidence was rated as low or very low because the level of evidence was downgraded to account for the variability in washout periods in the studies. The other reason for the downgrade was because of the small sample sizes of the studies (12 of 24 included studies had less than 20 participants each); hence, some of the MD from the meta-analysis was as little as 0.22 (0.03–0.47).

One of the major limitations of this review was that all the included studies had cross-over designs with different washout periods ranging from a few hours to 2 weeks between the treatment arms. The other limitation was that the risk of bias was high or unclear in the following domains: allocation concealment, blinding of participants, personnel and outcome assessors. Finally, some studies used different units of measurement and had to be converted to the units chosen for the study. While the authors ensured they used standard conversion formulae, it is possible some mistakes may still have occurred in the process of conversion. All the limitations listed have the potential to affect the eventual interpretation of the findings.

### Implications of the results

The findings of this review suggest that among the healthy adult population, surgical masks could be used safely for mild to moderate exercise but not vigorous exercises. The review also suggests that masks should be avoided in convalescing and elderly persons.

### Recommendations for future research

Future systematic reviews and meta-analyses may consider including more physiological and psychological outcomes. As more primary studies become available, more reviews should be conducted on children above the age of 5 years to generate evidence of mask use in them.

### Protocol registration

The protocol for this review was registered with PROSPERO ID=CRD42022356243 August 2022 (available from: https://www.crd.york.ac.uk/prospero/display_record.php?ID=CRD42022356243).
